# Characterization of polyvalent *Escherichia* phage Sa157lw for the biocontrol potential of *Salmonella* Typhimurium and *Escherichia coli* O157:H7 on contaminated mung bean seeds

**DOI:** 10.3389/fmicb.2022.1053583

**Published:** 2022-11-10

**Authors:** Yen-Te Liao, Yujie Zhang, Alexandra Salvador, Kan-Ju Ho, Michael B. Cooley, Vivian C. H. Wu

**Affiliations:** Produce Safety and Microbiology Research Unit, U.S. Department of Agriculture, Agricultural Research Service, Western Regional Research Center, Albany, CA, United States

**Keywords:** polyvalent phage, whole-genome sequencing, *Salmonella* Typhimurium, *Escherichia coli* O157:H7, alternative biocontrol agent, mung bean seeds

## Abstract

Seeds are one of the primary sources of contamination with foodborne pathogens, such as pathogenic *Escherichia coli*, and various *Salmonella* serovars, for produce, particularly sprouts. Due to the susceptibility of sprout growth to chemical-based antimicrobials and the rising issue of antimicrobial resistance, developing innovative antimicrobial interventions is an urgent need. Therefore, the objective of this study was to characterize *Escherichia* phage Sa157lw (or Sa157lw) for the biocontrol potential of *Salmonella* Typhimurium and *E. coli* O157:H7 on contaminated mung bean seeds. Phage Sa157lw was subjected to whole-genome sequencing and biological characterization, including morphology, one-step growth curve, and stress stability tests. Later, antimicrobial activity was determined *in vitro* and upon application on the mung bean seeds artificially contaminated with *E. coli* O157:H7 or *Salmonella* Typhimurium. Sa157lw possessed a contractile tail and belonged to the *Kuttervirus* genus under the *Ackermannviridae* family, sharing a close evolutionary relationship with *E. coli* phage ECML-4 and *Kuttervirus* ViI; however, tail spike genes (ORF_102 and ORF_104) were the primary region of difference. Comparative genomics showed that Sa157lw encoded a cluster of tail spike genes—including ORF_101, ORF_102, and ORF_104—sharing high amino acid similarity with the counterfeits of various *Salmonella* phages. Additionally, Sa157lw harbored a unique tail fiber (ORF_103), possibly related to the receptors binding of O157 strains. The genomic evidence accounted for the polyvalent effects of Sa157lw against *E. coli* O157:H7 and various *Salmonella* serovars (Typhimurium, Enteritidis, Agona, Saintpaul, and Heidelberg). Furthermore, the phage did not contain any virulence, antibiotic-resistant, or lysogenic genes. Sa157lw had a 30-min latent period on both *E. coli* O157:H7 and *Salmonella* Typhimurium, with an estimated burst size of 130 and 220 PFU/CFU, respectively, and was stable at a wide range of temperatures (4–60°C) and pH (pH4 to pH10). The phage application demonstrated a strong anti-*E. coli* O157:H7 and anti-*Salmonella* Typhimurium effects in 1.1 and 1.8 log reduction on the contaminated mung bean seeds after overnight storage at 22°C. These findings provide valuable insights into the polyvalent Sa157lw as a potential biocontrol agent of *Salmonella* Typhimurium and *E. coli* O157:H7 on sprout seeds.

## Introduction

Foodborne pathogens, such as *Salmonella* and Shiga toxin-producing *Escherichia coli* (STEC), have been causing an increasing number of foodborne outbreaks. More than 30% of these outbreaks are associated with various produce—alfalfa sprouts, iceberg lettuce, and melon—contributing to over 350 identified cases in the United States between 2018 and 2021 (Glaize et al., 2021). Although food contamination varies among different produce products, sprouts are the most affected by pathogenic *E. coli* and *Salmonella* strains, likely due to high humidity and organic compounds produced in the production system (Miyahira and Antunes, 2021). Based on the global epidemiologic data, an estimated 147,000 cases of sprouts-related foodborne outbreaks were reported from 1988 to 2020, causing 214 hospitalization and 58 deaths, primarily from *Salmonella* and STEC O157 infections (Miyahira and Antunes, 2021).

Sprout seeds are the primary contaminants for an enclosed sprout production line to contaminate the final sprout before packaging and distribution (Darmanin et al., 2021). Sprout seeds require warm and humid conditions for germination (NACMCF., 1999); macromolecules containing nutrient substances will be released during germination to facilitate the growth of surrounding bacteria (Yang et al., 2013). Moreover, skin-damaged seeds are more likely to be contaminated with bacterial pathogens than undamaged seeds and can even minimize the effectiveness of common antimicrobial agents in targeting the pathogens (Ding et al., 2013). In the sprout industry, calcium hypochlorite is used in standard practice to sanitize sprout seeds as it is more effective than non-chemical treatments, such as heat and irradiation (Riggio et al., 2019). Furthermore, electrolyzed oxidation water (EOW) is also used in produce sanitation because of its environmental safety, low cost, and easy preparation (Dewi et al., 2017). However, these antimicrobial interventions may interrupt the germination rate or adversely affect sprout quality during production (Bari et al., 2009; Fong et al., 2017); some could even cause bacterial resistance to hamper the efficacy of the treatment (Shu et al., 2020). Therefore, alternative methods are critically needed to ensure the microbiological safety of the seeds for sprout production without adversely impacting the quality (Mir and Kudva, 2019).

Though not new in the medical field, bacteriophage (or phage) application is a promising and innovative antimicrobial intervention in the agricultural field. Phages are bacteria-infecting viruses and are highly diverse and ubiquitous in the ecosystem where their bacterial hosts exist (O’Sullivan et al., 2019). Phages bind specifically to the receptor proteins on the membrane of the target bacterial cells to initiate the infection (Milho et al., 2019; Pinto et al., 2020). Due to the nature of the lytic cycle, phage infection always leads to the lysis of bacterial cells and the release of phage progenies for subsequent infection (Clokie et al., 2011). Unlike antibiotics, lytic phages are host-specific and do not wipe out unrelevant background flora that might be important to the environment (Kosznik-Kwaśnicka et al., 2022). Additionally, lytic phages can combat and reduce the spread of antibiotic-resistant strains in an agricultural field (Amarillas et al., 2016; Kahn et al., 2019) and minimize any environmental burden on subsequent terrestrial surroundings (O’Sullivan et al., 2019). These phages have been approved to be used in an organic farming system if isolated from natural sources without genetic modification (Endersen and Coffey, 2020). Most importantly, FDA has authorized several commercial phage products as Generally Recognized As Safe (GRAS) to ensure safe application directly on ready-to-eat food or during food production (Riggio et al., 2019; Vikram et al., 2020). Consequently, phage-based interventions provide a bright future both in the food industry and among consumers.

Numerous studies have evaluated the efficacy of lytic phages in mitigating bacterial pathogens on various produce. A previous study characterized a lytic phage ECP26 capable of infecting a wide range of STEC serogroups, O157 in particular; a 2.5-log reduction of viable *E. coli* O157:H7 cells was observed on romaine lettuce after phage treatment, following 4°C storage for 5 days (Park et al., 2020). Another study conducted by Snyder et al. (2016) also found that applying a lytic phage effectively controlled *E. coli* O157:H7 by more than 3 log reduction on contaminated spinach following storage at 4°C. Moreover, one study was conducted to determine the antimicrobial activity of phage SI1 against *Salmonella* Enteritidis (Fong et al., 2017). Their results showed phage SI1 decreased *Salmonella* levels on alfalfa seeds by 2.5 log CFU/g after phage treatment for 2 h at 22°C. Despite sprouts constantly being implicated in both *Salmonella* and STEC contamination (Miyahira and Antunes, 2021), the efforts concerning phage application focus more on the control of different *Salmonella* serovars rather than STEC O157. Thus, the information regarding the characterization of a lytic phage that can target both pathogenic *E. coli* and *Salmonella* is of significant interest but is scarce. Therefore, the objective of this study was to characterize a polyvalent *Escherichia* phage Sa157lw isolated from surface water for the biocontrol potential of *E. coli* O157:H7 and *Salmonella* Typhimurium on contaminated mung bean seeds.

## Materials and methods

### Phage isolation

*Escherichia* phage Sa157lw (or Sa157lw) was previously isolated with *E. coli* O157:H7 (ATCC 43888) from surface water in a produce-growing area (Liao et al., 2018). Phage propagation was performed by mixing 50 μl phage lysate (∼10^9^ PFU/ml) with 45 ml of the log-phase *E. coli* O157:H7 (ATCC 43888) culture in tryptic soy broth (TSB; Becton Dickinson, Sparks, MD, USA) and CaCl_2_ at 10 mM for incubation at 37°C for 20 h. The propagated phages were filtered through a 0.22-μm filter membrane, following centrifugation at 8,000 × *g* for 10 min before downstream analysis.

### Bacterial culture

A collection of the top 6 non-O157 STEC—the serogroups O26, O45, O103, O111, O121, O145—, *E. coli* O157:H7, generic *E. coli*, and *Salmonella enterica* strains were obtained from the Produce Safety and Microbiology (PSM) Research Unit at the U.S. Department of Agriculture (USDA), Agricultural Research Service (ARS), Western Regional Research Center, Albany, CA, United States for this study (Supplementary Table 1). *E. coli* O157:H7 (ATCC 43888) was used for phage propagation and quantification. Fresh bacterial culture was prepared by inoculating 10 ml TSB with 1 μl loopful of each strain for overnight incubation at 37°C before use.

### Genomic analysis

Phage Sa157lw was purified through a CsCl gradient and subsequently subjected to DNA extraction and library preparation before sequencing as previously described (Liao et al., 2022). Later, the samples were loaded to a MiSeq Reagent Kit v2 (500-cycle) and sequenced on the MiSeq platform (Illumina, San Deigo, CA, USA), generating approximately 14 million 2 × 250-bp paired-end reads. The updated annotation was generated using previously reported methods (Zhang et al., 2021) and deposited in National Center for Biotechnology Information (NCBI) database. Briefly, raw sequence reads were subjected to FASTQC and trimming via Trimmomatic with the setting of Q30. *De novo* assembly was conducted on the resulting quality reads using Unicycler Galaxy v0.4.6.0 (SPAdes v2.5.1), followed by annotation via the Prokka pipeline Galaxy 1.13 (Seemann, 2014) with the default settings. Subsequently, the annotation was manually curated with Universal Protein Resource (UniProt) database (Bairoch et al., 2005) using Geneious (v11.0.3, Biomatters, New Zealand). tRNAscan-SE (v2.0) server was used to confirm the predicted tRNAs in the phage genome (Lowe and Chan, 2016).

Genome-BLAST Distance Phylogeny (GBDP) method (Meier-Kolthoff et al., 2013) was used to determine the whole-genome phylogenetic evolution at amino acid levels via the Virus Classification and Tree Building Online Resource (VICTOR) between Sa157lw and the reference phages belonging to the *Ackermannviridae* family, with high sequence similarity to Sa157lw based on NCBI blastn results. The visualization of genome comparison between Sa157lw and its closely related reference phages was conducted using the EasyFig v2.2.5 (Sullivan et al., 2011). Comparative analysis was conducted on amino acid sequences of the ORFs coding for tail spike proteins (ORF_102, ORF_103, and ORF_105), putative tail fiber (ORF_104), lysozyme (ORF_197), and terminase large subunit (ORF_114) between Sa157lw and the close-related reference sequences from the NCBI Protein Reference Sequences database.^1^ The amino acid sequences were aligned using the ClustalW (version 1.2.3). Then, the phylogenetic tree was generated using the MEGA11 program with the maximum composite likelihood method and 500 bootstrap replications (Tamura et al., 2021) and further annotated using the Interactive Tree of Life (iTOL) webserver (Letunic and Bork, 2019). Because no phage DNA packaging mechanism was predicted via the PhageTerm tool (Garneau et al., 2017), the reference terminase large subunit genes, previously experimented with in other studies for various packaging mechanisms, were used for the phylogenetic analysis (Supplementary Table 2). The presence of antibiotic-resistance genes and virulence genes in the phage genome was examinated using the ResFinder 4.1 webserver (Florensa et al., 2022) and VirulenceFinder (Malberg Tetzschner et al., 2020).

### Transmission electron microscopy

Phage Sa157lw was purified using a CsCl gradient, and 5 μl of the phage was placed on a discharged 300 mesh carbon/formvar coated grid to absorb for 2 min. After washing with distilled water and removing extra liquid, 5 μl of 1% uranyl acetate stain was added to the grid for negative staining, followed by drying. The specimen was then examined under a transmission electron microscope FEI Tecnai 12 120kV TEM (FEI, Hillsboro, OR, USA).

### Host range and efficiency of plating determination

The host range test for Sa157lw was conducted against non-pathogenic *E. coli*, *E. coli* O157:H7, top six non-O157 STEC, and various *Salmonella* strains using a spot test assay as previously described (Liao et al., 2019). The phage-sensitive strains were further used to determine productive infection of phage Sa157lw versus the phage progenies produced from the primary host strain (ATCC 43888) using the efficiency of plating (EOP) assay described in a previous study (Liao et al., 2011). Briefly, fresh cultures were prepared in TSB at 37°C overnight for quantifying Sa157lw using the double-layer plaque assay with diluted phage lysate of four successive dilutions (10^–3^ to 10^–7^). The plates were incubated at 37°C for 18 h. The experiment was conducted in three replications. Generally, a high phage-producing efficiency had EOP of 0.5 or more; a medium-producing efficiency had EOP above 0.1 but below 0.5; a low-producing efficiency had EOP between 0.001 and 0.1; inefficient phage production was any value lower than 0.001.

### One-step growth curve

One-step growth curve of phage Sa157lw was conducted separately on *E. coli* O157:H7 (ATCC 43888) and *Salmonella* Typhimurium (ATCC 14028) based on the previous method with subtle modification (Liao et al., 2022). In brief, the fresh culture of each bacterial strain was prepared in 10 ml of TSB at 37°C for 20 h; 0.2 ml of the overnight culture was sub-cultured in 19.8 ml of TSB and incubated for 2 h at 37°C to reach the log phase of the bacterial growth. Subsequently, phage Sa157lw was added to the log-phase bacterial solution (MOI of 0.01) with CaCl_2_ at 10 mM and incubated at 37°C for 15 min to allow phage adsorption to bacterial cells. The phage-bacterial mixture was centrifuged at 10,000 × *g* for 5 min at 4°C and the supernatant was discarded. Later, the bacterial pellet was washed with 2 ml TSB and subsequently resuspended in 20 ml of fresh TSB. After homogenization, 0.3 ml resuspended culture was added to 29.7 ml of TSB and incubated at 37°C with shaking throughout the experiment (75 min for both *Salmonella* and *E. coli*). Meanwhile, quantification of phage-infected bacteria was determined at time 0 by mixing 50 μl of the 30-ml phage-bacterial mixture (no filtration) with 100 μl of fresh overnight bacterial culture and 5 ml of molten 50% tryptic soy agar (TSA) for double-layer plaque assay. Additionally, 1 ml of the sample was obtained from the 30-ml phage-bacterial mixture every 5 min and subjected to 0.22-μm membrane filtration. The titers of Sa157lw were determined at each time point using a double-layer plaque assay, and plaque assay plates were incubated at 37°C overnight. The experiment was conducted in three replications to estimate the latent period and burst size of phage Sa157lw.

### Stability tests for pH, temperature, and refrigeration storage

A range of pH values from pH3 to pH12 were used to test the stability of Sa157lw at 25°C for 24 h using the method as previously described with minor changes (Liao et al., 2022). In brief, 100 μl of phage Sa157lw was added to 5 ml of SM buffer with final pH values of 3, 4, 5, 7.5, 10, and 12 and incubated at 25°C for 24 h. Viable phage particles of Sa157lw were determined with *E. coli* O157:H7 (ATCC 43888) using the double-layer plaque assay.

To determine temperature stability of Sa157lw, a bulk phage solution was prepared by mixing the original phage lysate with SM buffer at 1:9 (v/v) ratio. Later, an aliquot of 1 ml Sa157lw solution per tube was dispensed in several sterile microcentrifuge tubes and subjected to various temperatures, including 22, 30, 40, 50, 60, and 65°C, for 24 h. The temperatures covered common and extreme conditions in food-related environments. Phage titers were determined using the double-layer plaque assay.

For storage stability at refrigeration temperature, a bulk phage solution was prepared by mixing original phage lysate with SM buffer and dispensed with 500 μl phage solution per tube in microcentrifuge tubes. The phages were stored at 4°C for 90 days. Phage titers were determined on day 0 (before refrigeration storage), day 7, day 14, day 30, day 45, day 60, day 75, and day 90 using the double-layer plaque assay. All experiments were conducted in three replications.

### Bacterial challenge assay of Sa157lw

The bacterial challenge assay was performed to measure the effects of phage Sa157lw with different MOIs on bacterial growth based on bacterial optical density at a wavelength of 600 nm (OD_600_) as previously described with minor change (Zhang et al., 2021). In brief, fresh bacterial culture of *E. coli* O157:H7 (ATCC 35150) or *Salmonella* Typhimurium (ATCC 14028) was prepared in TSB at 37°C overnight and further diluted in TSB to 1 × 10^6^ CFU/ml. An aliquot of 200 μL diluted bacterial solution per well was added to a 96-well plate. Later, 10 μL of phage Sa157lw with different titers was added to the wells to reach MOIs of 1, 10, and 100; the control group contained only bacterial solution without phage. The reading of OD_600_ was measured using a spectrophotometer (Promega, Madison, WI, USA) at 25°C every 30 min for 12 h. The experiment was conducted in three replications.

### Lysis from without

Bacterial cell lysis without a complete phage infection, also known as lysis from without (LO), was determined on *E. coli* O157:H7 (ATCC 35150) and *Salmonella* Typhimurium (ATCC 14028) strains treated with different MOIs of phage Sa157lw using a spectrophotometer with minor modification (Liao et al., 2022). In brief, fresh overnight culture was prepared in 10 ml TSB and diluted in TSB to reach a final concentration of approximately 1 × 10^5^ CFU/ml. The diluted bacterial solution was dispensed onto a 96-well plate with 180 μl per well, followed by adding 20 μl of phage lysate (diluted in SM buffer) at the MOIs of 1, 10, and 100, with 3 wells per MOI. Three wells containing bacterial culture with 20 μl SM buffer without the phage were served as a control group. The OD_600_ was measured after 5-min incubation at room temperature. Three replications were conducted for statistical analysis.

### Phage application on mung bean seeds

A batch of mung beans seeds obtained from a local store was disinfected by 2% of NaClO solution for 15 min, followed by rinsing with sterile water five times and air drying at room temperature under a biosafety hood as previously described (Liao et al., 2022). Later, 5 g of the sterile mung bean seeds were homogenized with 20 ml 0.1% peptone water and plated on Sorbitol MacConkey (SMAC; Oxoid, Basingstoke, United Kingdom) and xylose lysine deoxycholate (XLD; Oxoid, Basingstoke, United Kingdom) plates to check if the seeds were free of *E*. *coli* and *Salmonella*.

Bacterial inoculums were prepared by mixing 0.2 ml of the overnight culture of *E. coli* O157:H7 (ATCC 35150) or *Salmonella* Typhimurium (ATCC 14028) with 40 ml sterile LB broth prior to mung bean seed inoculation. Seventy grams of seeds were submerged in the bacterial solution for 1 h at room temperature and air dried in a biosafety hood before refrigeration storage at 4°C overnight. The next day, bacterial levels on the inoculated mung bean seeds were determined by mixing 10 g of the inoculated seeds with 20 ml 0.1% peptone water for 2 min and spread plating on the selective media (SMAC or XLD plates) for incubation at 37°C overnight. Meanwhiles, the inoculated seeds (20 g) were immersed in phage Sa157lw solution (in SM buffer) at an MOI of approximately 6,000 for 1 h at room temperature. The seeds immersed in the same volume of 0.1% peptone water served as a control. Both treatment and control groups were stored at 22°C for 20 h before bacterial quantification. Later, 10 g of control and treatment seeds were homogenized with 20 ml 0.1% peptone water, followed by serial dilution and plating on SMAC or XLD overlayered thin TSA (Thin Agar Layer Method, TAL) (Wu, 2008). The plates were subjected to overnight incubation at 37°C.

### Determination of bacteriophage-insensitive mutant

Bacterial colonies of *E. coli* O157:H7 or *Salmonella* Typhimurium recovered from the treated mung bean seeds were obtained from the selective media (SMAC TAL and XLD TAL plates, respectively). The bacteriophage-insensitive mutants (BIMs) were determined on the selected colonies using a spectrophotometer (Fong et al., 2017). In brief, the original bacteria (*E. coli* O157:H7 or *Salmonella* Typhimurium) and the bacteria obtained from the treated seeds were incubated in TSB overnight at 37°C. Each bacterial culture was added to four wells in a 96-well plate, with two wells added with phage Sa157lw at an MOI of 1. Later, the 96-well plate was incubated at 30°C overnight prior to measuring the optical density at 600 nm (OD_600_). The BIM was confirmed if the OD_600_ value of the wells with phage was similar to those without phage. The original bacterial culture was used as a control set for comparison.

### Statistical analysis

Experiments subjected to statistical analysis were conducted in three individual replications. The quantification of bacteria and phages was calculated as CFU/g and PFU/ml, respectively, with logarithmical conversion for statistical analysis. The stress effects of pH (pH4 to pH10), temperature, and storage on phage Sa157lw were determined using a one-way analysis of variance (ANOVA) with a statistical significance at a 5% level. The Student’s *t*-test was used to analyze the antimicrobial activity of Sa157lw between the control and treatment groups on the mung bean seeds contaminated with *E. coli* O157:H7 or *Salmonella* Typhimurium.

## Results

### Genomic and comparative analyses of Sa157lw

**FIGURE 1:**
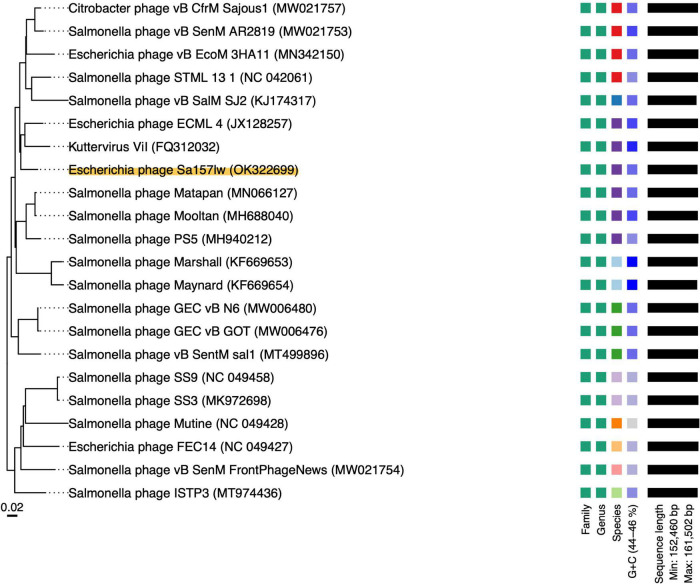
Phylogenetic analysis of whole-genome sequences of Sa157lw and close-related reference phages belonging to the *Kuttervirus* genus under the *Ackermannviridae* family at the amino acid level using VICTOR (formula d6) (https://ggdc.dsmz.de/victor.php). Annotations, including family, genus, and species cluster proposed by VICTOR, genomic G + C content and sequence length, are given to the right-hand site of the tree. The accession number of each phage is provided in the parenthesis.

**FIGURE 2:**
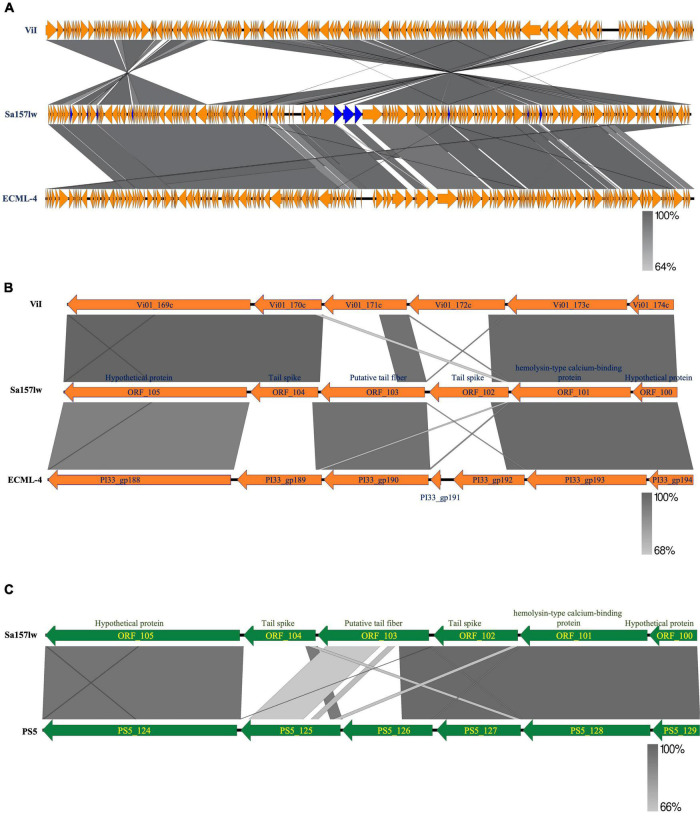
Genome comparison of the whole genome of Sa157lw and its close-related *Kuttervirus* ViI and *E. coli* phage ECML-4 **(A)**, the sequence region of tail fiber and tail spike **(B)**, and the same gene cluster between phages Sa157lw and PS5 **(C)** using EasyFig v2.2.5. Whole genome maps are presented as orange arrows, indicating the order of annotated ORFs from left to right along the phage genomes. The sequence regions of the tail-related gene cluster are presented as orange or green arrows. Regions of sequence similarity are connected by a gray-scale shaded area. The unshared ORFs between Sa157lw and ViI or ECML-4 are in blue. The sequence direction is adjusted for comparing the regional sequence.

Phage Sa157lw had double-stranded DNA with 155,887-bp genome size and an average GC content of 44.9%. The evolutionary tree of whole-genome sequence based on the VICTOR results showed that Sa157lw shared close relation with *Escherichia* phage ECML-4 (Carter et al., 2012) and *Kuttervirus* ViI (also known as *Salmonella* phage Vi01) (Adriaenssens et al., 2012) at the amino acid level among 21 reference phages belonging to the *Kuttervirus* genus (Figure 1). Moreover, VICTOR analysis predicted that phages Sa157lw, ECML-4, and ViI were at the same species level as *Salmonella* phages Matapan, Mooltan, and PS5 from another clade. Phage PS5 was able to infect both *Salmonella* and *E. coli* O157:H7 (Duc et al., 2020). Comparative analysis showed that Sa157lw lacked common regions, including ORF_9, ORF_15, ORF_19, ORF_20, ORF_32, ORF_84, ORF_102, ORF_103, ORF_104, ORF_128, ORF_155, ORF_156, and ORF_ 161, between either *Escherichia* phage ECML-4 or *Kuttervirus* ViI (Figure 2A). Except for most ORFs with hypothetical functions and missing ORF_9, coding for putative HNH endonuclease, in phage ViI, the primary differences between Sa157lw and both ViI and ECML-4 were in the region containing ORF_102, ORF_103, and ORF_104 that encoded tail pike, putative tail fiber, and tail spike protein, respectively (Figure 2B). The results revealed that ORF_104 was likely associated with *E*. *coli* O157:H7-binding affinity, while ORF_103 was associated with the binding ability of *Salmonella* strains. Compared to the similar region in phage PS5, Sa157lw did not share a common nucleotide sequence identity of ORF_104 (Figure 2C).

**FIGURE 3:**
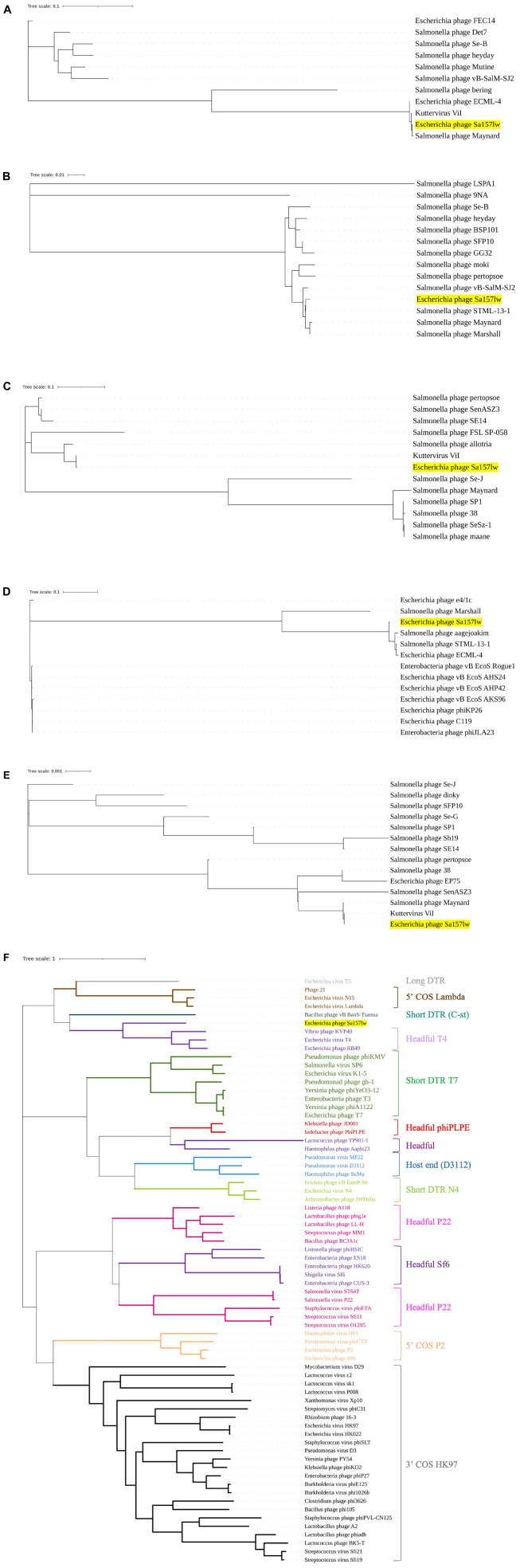
Neighbor-joining phylogenetic tree of phage Sa157lw (with yellow highlight) and the reference phages belonging to the *Kuttervirus* genus based on the Clustal Omega alignment of the amino acid sequences of hemolysin-type calcium-binding protein (ORF_101) **(A)**, tail spike protein (ORF_102) **(B)**, tail spike protein (ORF_104) **(C)**, putative tail fiber protein (ORF_103 **(D)**, tail lysozyme (ORF_196) **(E)**, and terminase large subunit **(F)**. The scale represents the homology percentage.

Phage Sa157lw contained 210 ORFs, including 64 annotated with predicted functions and 4 tRNAs (Ser, Asn, Met, and one undetermined). The ORFs annotated as known functions were associated with structural proteins, host lysis (lysozyme and peptidoglycan binding protein), phage DNA replication, transcription regulation, host cell regulation, and metabolism (Supplementary Table 3). Additionally, *stx* genes, lysogenic genes, and antibiotic resistance genes were absent in the genome of Sa157lw. Phylogenetic analyses were conducted on the ORFs encoding the proteins associated with bacterial host recognition, bacterial cell lysis, and phage replication mechanism. Sa157lw contained three tail spike proteins, encoded by ORF_101 (annotated with hemolysin-type calcium-binding protein), ORF_102, and ORF_104, sharing a close evolutionary relationship at the amino acid level with the counterfeit in *Salmonella* phage Maynard, *Salmonella* phage STML-13-1, and *Kuttervirus* ViI, respectively (Figures 3A–C). Moreover, Sa157lw contained ORF_103 encoding a unique tail fiber, which did not share the evolutionary relationship with any reference phages (Figure 3D). All these four ORFs were associated with the capability of Sa157lw to infect both *Salmonella* and the O157 serogroup of STEC strains. For tail lysozyme (cell lysis function), Sa157lw shared a high amino acid identity with the counterpart of *Kuttervirus* ViI (Figure 3E). Concerning the phage DNA packing mechanism, Sa157 encoded a terminase large subunit sharing a high sequence similarity at amino acid levels to the phages classified as Headful T4 (Figure 3F).

### Phage morphology

**FIGURE 4:**
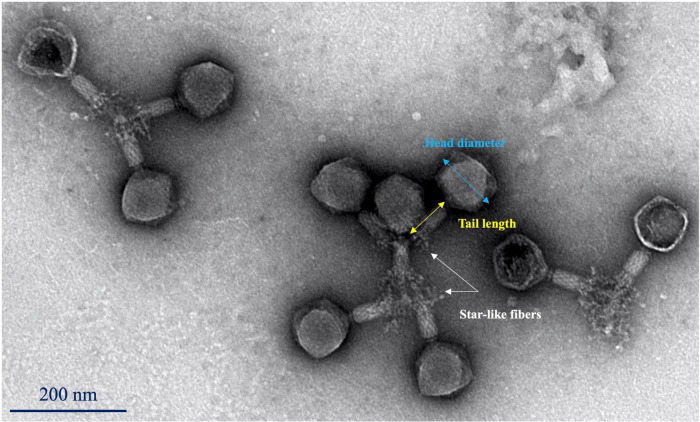
Transmission electron microscopy image of phage Sa157lw with an icosahedral capsid (99 ± 3 nm in diameter) and a contractile tail (87 ± 3 nm in length).

Phage Sa157lw had an icosahedral head of approximately 99 ± 3 nm in diameter, and a long contractile tail of 87 ± 3 nm in length (Figure 4). The phage had a visible neck and star-like tail fibers at the end of the tail sheath structure, similar to the morphology of *Kuttervirus* ViI (Adriaenssens et al., 2012). Additionally, the phages seemed to be at contracted status, showing the *Myoviridae* morphology.

### Host range and productive infection

Phage Sa157lw was a polyvalent phage able to infect various *S. enterica* serovars—including Heidelberg, Saintpaul, Agona, Typhimurium, and Enteritidis—and *E. coli* O157:H7 strains (Table 1). Further examination of productive infection of Sa157lw on the susceptible bacteria was determined in comparison to the primary host *E. coli* O157:H7 strain (ATCC 43888) using the EOP assay. The results showed that various *E. coli* O157:H7 strains had a medium to high phage-producing efficiency, ranging from 0.33 to 0.94, and all susceptible *S. enterica* serovars, except for *Salmonella* Agona, had high phage-producing efficiencies of >1 (Table 1).

**TABLE 1:** Host range and efficiency of plating of phage Sa157lw against various *Salmonella enterica* serovars and *E. coli* O157:H7 strains.

Strains	Strain reference number	EOP*
Non-O157 STEC	STEC O26, O45, O103, O111, O121 and O145	R
STEC O157	*E. coli* O157:H7 (RM18959)	0.8
	*E. coli* O157:H7 (ATCC 35150)	0.82
	*E. coli* O157:H7 (ATCC 43888)	H
	*E. coli* O157:H7 (RM 9995)	0.33
	*E. coli* O157:H7 (RM 6416)	0.44
	*E. coli* O157:NM (RM 11781)	0.94
Generic *E. coli*	ATCC 13706	R
	DH5a	R
*Salmonella*	*Salmonella* Montevideo S1	R
	*Salmonella* Newport H1073	R
	*Salmonella* Heidelberg 45955	Inefficiency
	*Salmonella* Enteritidis PT-30	1.2
	*Salmonella* Typhimurium 14028	1.6
	*Salmonella* Agona (W0081)	0.14
	*Salmonella* Anatum (W0082)	R
	*Salmonella* Enteritidis H3527 (W0100)	1.1
	*Salmonella* Saintpaul 39 (W0106)	Inefficiency
	*Salmonella* Thompson 132 (W0107)	R

*EOP was calculated by the ratio of phage titer on a test bacterium versus the primary bacterial host.

High production efficiency is EOP ≥ 0.5, medium production efficiency is 0.5 > EOP ≥ 0.1, low production efficiency is 0.1 > EOP > 0.001, and the inefficiency of phage production is EOP ≤ 0.001.

H means the host strain used for the phage isolation. R means the bacterial strain is resistant to the phage infection.

### One-step growth curve and stability tests

**FIGURE 5:**
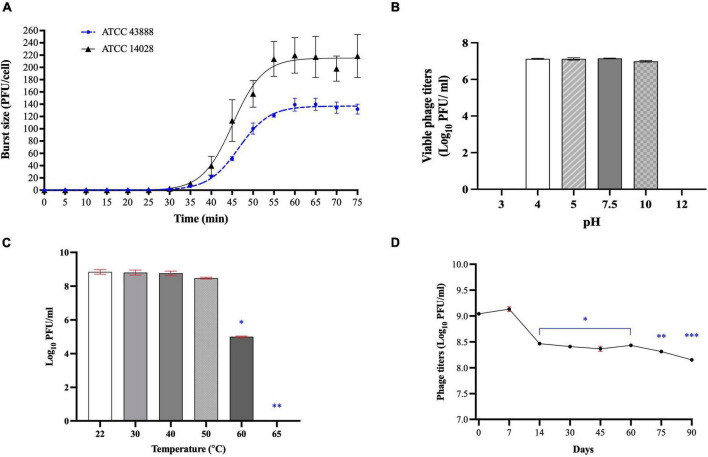
Biological characteristics of phage Sa157lw, including one-step growth curve on both *E. coli* O157:H7 ATCC 43888 and *Salmonella* Typhimurium ATCC 14028 **(A)**, various pH stability at 25°C for 24 h **(B)**, temperature stability for 24 h **(C)**, and refrigeration storage at 4°C for 90 days **(D)**. For stability tests, means of phage titers that contain different numbers of asterisks differ (*P* < 0.05). The error bars show the SEM.

Phage Sa157lw had a 30-min latent period against *E. coli* O157:H7 ATCC43888 and *Salmonella* Typhimurium ATCC14028 (Figure 5A). An average burst size of 130 ± 18 and 220 ± 50 progeny virions per infected cell was determined on *E. coli* O157:H7 and *Salmonella* Typhimurium, respectively, after a 75-min incubation at 37°C.

For pH tolerance, the phage was able to maintain the titers of 7.1 log PFU/ml between pH4 and pH10 for 24 h at 25°C but significantly dropped (*P* < 0.05) to a non-detectable level at both pH3 and pH12 (Figure 5B). Regarding temperature stability, phage Sa157lw was stable at temperatures ranging from 22 to 50°C after 24-h treatment but decreased in titer at 60°C by approximately 3 log and dropped to a non-detectable level at 65°C (Figure 5C). During the refrigeration storage, phage titers of Sa157lw decreased significantly after storage for 14 days at 4°C by approximately 0.5 log and subsequently maintained for 60 days of storage before further dropping 0.2 log (*P* < 0.05) after 90 days (Figure 5D).

### Lysis from without

Bacterial lysis without a complete phage infection, also known as lysis from without (LO), was determined on *E. coli* O157:H7 (ATCC 35150) and *Salmonella* Typhimurium (ATCC 14028) with phage Sa157lw at different MOIs (1, 10, and 100) using a spectrophotometer. The results showed that LO was observed on *E. coli* O157:H7 treated with phage at the MOI of 100 due to the low OD_600_ (*P* < 0.05) in compassion to lower concentrations of phage (MOIs of 1 and 10) and the control group (Supplementary Figure 1). Though not a significant difference, MOI of 100 resulted in the lowest OD_600_ value among all MOIs for *Salmonella* Typhimurium.

### Bacterial challenge assay of Sa157lw

**FIGURE 6:**
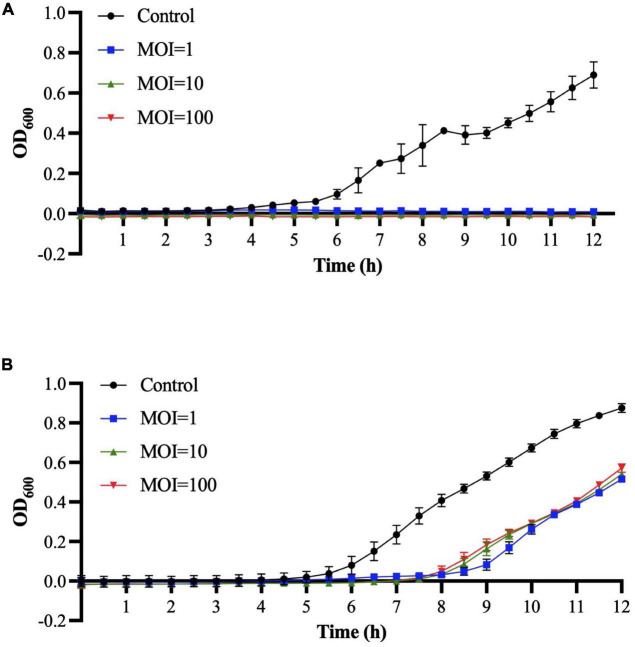
Bacterial challenge assay for *E. coli* O157:H7 **(A)** and *Salmonella* Typhimurium **(B)** treated with phage Sa157lw at MOIs of 1, 10, and 100 at 25°C for 12 h based on the bacterial optical density at wavelength 600 nm (OD_600_). The control group only contains bacterial culture.

The *in vitro* antimicrobial activities of Sa157lw at MOIs of 1, 10, and 100 were determined against *E. coli* O157:H7 (ATCC 35150) and *Salmonella* Typhimurium (ATCC 14028) at 25°C using a spectrophotometer. The results showed that the growth of *E. coli* O157:H7 was completely inhibited during the 12-h incubation among all MOIs (Figure 6A). For *Salmonella* Typhimurium, the bacteria treated with Sa157lw, regardless of MOIs, started to grow after 8 h of incubation at a similar pattern (Figure 6B).

### Application of Sa157lw on the contaminated mung bean seeds

**FIGURE 7:**
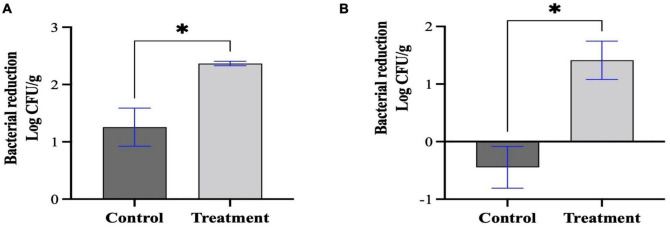
Bacterial reduction of *E. coli* O157:H7 **(A)** and *Salmonella* Typhimurium **(B)** treated by Sa157lw at an MOI of approximately 6,000 for 1 h at room temperature. Both control (bacteria only) and treated mung bean seeds were stored at 22°C for 20 h prior to bacterial quantification. The initial inoculation levels on the mung bean seeds for *E. coli* O157:H7 and *Salmonella* Typhimurium were 2.36 and 2.86 log CFU/g, respectively. Asterisk indicates a significant difference at *P* < 0.05 between the control and treatment groups.

Based on the bacterial challenge assay results, Sa157lw at low MOIs (1, 10, and 100) had similar bacterial inhibition effects. Thus, a high MOI of approximately 6,000 was utilized to increase the chance of contact between phage Sa157lw and the target bacterial cells. The results showed that *E. coli* O157:H7 (ATCC 35150) was reduced by 1.1 ± 0.6 log in comparison to the control group (free of phage) (a total of 2.4 ± 0.1-log reduction) after 1-h phage treatment at room temperature, followed by storage at 22°C overnight (Figure 7A). For *Salmonella* Typhimurium, the treatment group had a 1.9 ± 1-log reduction of the bacterial cells compared to the control group (Figure 7B). Additionally, the results revealed that the levels of *E. coli* O157:H7 on the seeds without phage treatment dropped, but the viable cells of *Salmonella* Typhimurium without phage increased during the overnight storage period.

Phage resistance was assessed from the bacteria recovered from the treated mung bean seeds compared to the wild-type bacterial strains using a spectrophotometer. The results showed that two out of 14 *E. coli* O157:H7 colonies were resistant to Sa157lw infection, with similar OD_600_ values between phage-treated and untreated cultures; for *Salmonella* Typhimurium, there were two out of 22 colonies developing phage resistance (results not shown).

## Discussion

Lytic phages are alternative antimicrobials to control bacterial pathogens due to the enhanced development of antimicrobial resistance from the current chemical interventions (Moye et al., 2020). After the FDA approved the first lytic phage product as GRAS in 2006 (Kahn et al., 2019), phage-based interventional technology sheds new light on developing green, cost-effective, and potent antimicrobial intervention methods to improve food safety. In this study, we characterized a new polyvalent phage Sa157lw via biological and genomic characterization for the biocontrol potential of both *E. coli* O157:H7 and *Salmonella* Typhimurium on artificially contaminated mung bean seeds.

Phage Sa157lw is a new member of phages with a contractile tail belonging to the genus *Kuttervirus*, subfamily *Cvivirinae*, and family *Ackermannviridae*. Comparing 20 reference phages (17 *Salmonella* and 3 *E. coli* phages), Sa157lw has a high sequence similarity at the amino acid level with *E. coli* phage ECML-4 and *Kuttervirus* ViI (also known as *Salmonella* phages Vi01), all belonging to the genus *Kuttervirus*. Previous studies found that the genus *Kuttervirus* was composed of the phages that infected either *Salmonella* or *E. coli* (Fan et al., 2021). The host range spectrum was primarily influenced by a cluster of tail spike genes, each recognizing a specific receptor of their hosts (Sørensen et al., 2021). Based on the current results of comparative genomics, Sa157lw contains two tail spike-encoding genes (ORF_102 and ORF_104) that are highly associated with recognizing the receptor proteins on *Salmonella* membranes and a putative tail fiber-encoding gene (ORF_103), related to the infection of *E. coli* O157:H7 strains. Additionally, Sa157lw has a different *Salmonella* host range from those of phages ViI and PS5, primarily due to the nucleotide sequence diversity of ORF_102. Sa157lw also encodes a tail lysozyme sharing a high amino-acid similarity to *Kuttervirus* ViI. This protein may contribute to a strong affinity of Sa157lw toward several *Salmonella* serovars due to its C-terminal domain, which is associated with the recognition and adsorption of bacterial lipopolysaccharide on the cell membrane (Bhardwaj et al., 2014). Furthermore, Sa157lw harbors a unique tail fiber-encoded gene adjacent to the cluster of tail spike genes that could be one of the primary factors for the phage to infect *E. coli* O157:H7. Sa157lw also contains four tRNAs located near the cluster of tail spike genes that are likely associated with the enhancement of tail structure protein expression to infect more bacteria, increasing fitness to various environmental stresses (Delesalle et al., 2016; Liao et al., 2019, 2022). These findings support Sa157lw’s polyvalent attributes against a broad range of bacteria, including *E. coli* O157:H7, *S.* Typhimurium, *S.* Enteritidis, *S.* Agona, *S.* Heidelberg, and *S.* Saintpaul, unlike other *Kuttervirus* phages that only target either *Salmonella* or *E. coli* species (Sharma et al., 2009; Hooton et al., 2011; Fan et al., 2021). Sa157lw does not contain *stx* genes, antibiotic-resistance genes, or any lysogenic genes, thus suggesting the safety of the antimicrobial agent for application.

The characterization of the phage population growth shows that Sa157lw has the same latent period (30 min) but different burst sizes against *E. coli* O157:H7 (130 PFU/CFU) and *Salmonella* Typhimurium (220 PFU/CFU), similar to that of another polyvalent phage PS5 isolated from chicken (Duc et al., 2020). A previous study showed that the holin protein expressed by a lytic phage was the primary factor affecting the latent period due to its role in causing membrane lesions and allowing endolysin to attack murein on the cell wall (Young et al., 2000; Abedon et al., 2001). Subsequently, bacterial cells were lysed to release phage progenies in the environment. However, the holin-encoded gene was not found in the genome of Sa157lw nor in the closely related phages ECML-4 and *Kuttervirus* ViI. Thus, the regulatory factors contributing to the holin-like function require further investigation. Generally, Sa157lw has large burst sizes infecting either *E. coli* O157:H7 or *Salmonella* Typhimurium. A lytic phage with a large burst size is advantageous in combating the target bacteria because significant numbers of phage progenies will be produced for diffusion and subsequent infection (Nilsson, 2014). Phage stability also plays a significant role for phages in active status to target their bacterial host upon application under various environmental factors. Sa157lw demonstrates stability in maintaining infectivity from pH4 to pH10, a pH range most *E. coli*-infecting phages can also sustain (Merabishvili et al., 2013; Son et al., 2018; Liao et al., 2022). For thermal susceptibility, phage Sa157lw can withstand the treatment of 60°C for 24 h and remain stable at 4°C for at least 90 days. These biological characteristics of Sa157lw are sufficient to withstand typical produce-associated environments.

Upon phage application, a high MOI is preferable to increase the probability of phage contact with the target bacteria in a food-associated environment, and the resulting LO may provide extra mitigating effects. The current results demonstrate a 2.4-log reduction of *E. coli* O157:H7 on the phage-treated seeds with the synergistic effects of overnight storage at 22°C; however, unlike *Salmonella* Typhimurium, the viable cells of *E. coli* O157:H7 on the control seeds without phage treatment also dropped. As a result, phage treatment with MOI of 6,000 alone reduces more *Salmonella* Typhimurium than *E. coli* O157:H7 on the contaminated seeds. Although the 2.4-log reduction by the phage application is lower than the level suggested by the U.S. Environmental Protection Agency (EPA) protocol, phage application is still promising but requires further efforts to improve the antimicrobial efficacy and public acceptance. Additionally, the current results reveal that MOI of 100 does not cause LO on *Salmonella* Typhimurium but *E. coli* O157:H7, indicating high cell wall stability of the *Salmonella* strain (Abedon, 2011). The evidence likely suggests *Salmonella* Typhimurium used in this study is more resilient than *E. coli* O157:H7 to the non-phage environmental stress. Although dryness on the surface of the treated seeds may hinder phage movement and thus minimize the antimicrobial activity (Liao et al., 2022), phages can maintain viability at a prolonged period of storage (Park et al., 2020). As a result, the lytic effect of phage could subsequently continue bacterial killing in a hydrated/humid environment. Moreover, contrary to the treatment of freshly inoculated mung bean seeds (Liao et al., 2022), the bacterial-inoculated seeds in the current study were stored at refrigeration temperature for at least 1 day before the phage application experiment. The bacterial pathogens subjected to storage conditions may subsequently adjust their growth factors to survive through the carbon starvation conditions (Biselli et al., 2020), thus adversely affecting phage adsorption efficiency for successful lytic infection.

The information concerning phage application on sprouts for STEC O157, in particular, is scarce as compared to other produce, and the antimicrobial activities, in general, vary among studies (Yang et al., 2013; Fong et al., 2017; Zhang et al., 2019). A previous study conducted by Fong et al. (2017) isolated and characterized a new phage for the potential biocontrol of *Salmonella* on contaminated sprouting alfalfa seeds. The authors found that a single potent phage significantly decreased non-typhoidal *Salmonella* on day 1 of post-phage treatment; however, the viable bacterial levels remained stagnant throughout the 5-day storage period. On the contrary, another study demonstrated that a phage cocktail was not effective in mitigating *Salmonella* on adulterated sprout seeds or sprouts from the adulterated seeds (Zhang et al., 2019). The phenomenon is primarily due to the nature of the surface of sprout seeds or sprouts present with organic materials that compromise the effectiveness of phage application (NACMCF., 1999) and nutrient substances produced during germination that facilitate bacterial growth (Yang et al., 2013). The results of the current study also demonstrated no lytic effects of phage Sa157lw during the germination of the contaminated mung bean seeds (data not shown). Therefore, combining another method to reduce the interference of the organic materials during sprout production along with phage application is worth trying to improve the microbiological safety of sprout.

In this study, potential phage-resistant *Salmonella* and *E. coli* O157:H7 were found from the contaminated mung bean seeds treated with phage Sa157lw at an MOI of ∼6,000; the percentage of the resistant strains developed was slightly higher than the results in our previous study (Liao et al., 2022). The situation could be due to a high MOI used for the phage application that causes selection pressure (Necel et al., 2021). Phage resistance is a naturally occurring event and is a mechanism bacteria adopt to prevent phage infection by altering the outer bacterial membrane protein (OMP) and lipopolysaccharide (LPS), leading to phage adsorption failure (Bertozzi Silva et al., 2016; Rostøl and Marraffini, 2019). However, it is not always undesirable for bacteria to develop phage resistance in exchange for a fitness cost, which often causes decreased bacterial growth rate (Burmeister and Turner, 2020), loss of virulence (Wang, 2002), or higher sensitivity to environmental stress (Smith et al., 2007). Consequently, these strains will be susceptible to the subsequent non-phage interventions or treatment of different phages; thus, a hurdle intervention method or a phage cocktail could be the solution to improve the efficacy and reduce the MOI used for phage application (Zhang et al., 2019, Moye et al., 2020).

Overall, this study provides valuable insight into genomic and biological features of lytic phage Sa157lw exhibiting strong antimicrobial effects against both *E. coli* O157:H7 and various *Salmonella* serovars, including Typhimurium, Enteritidis, and Agona. With high external stress stability and large burst sizes against both *E. coli* and *Salmonella* species, the polyvalent phage Sa157lw is a promising biocontrol agent to prevent the contamination of these foodborne pathogens on sprout seeds. Additionally, these characteristics allow Sa157lw to be used as a hurdle intervention or as a cocktail formulation to improve the efficacy of phage application.

## Data availability statement

The datasets presented in this study can be found in online repositories. The names of the repository/repositories and accession number(s) can be found in the article/Supplementary material.

## Author contributions

Y-TL performed the phage isolation, one-step growth curve, bacterial challenge assay, lysis from without, data analyses, genomic analysis, and manuscript preparation. YZ and AS performed the whole-genome sequencing. K-JH performed the stability tests, one-step growth curve, and bacterial reduction experiments. MC edited the manuscript. VW conceived and supervised the study, aided in experiment design, and edited the manuscript. All authors reviewed the manuscript.

## Footnotes

1
https://www.ncbi.nlm.nih.gov/refseq/
