# Supplementary material for: Characterization of polyvalent Escherichia phage Sa157lw for the biocontrol potential of Salmonella Typhimurium and Escherichia coli O157:H7 on contaminated mung bean seeds
Source: Front Microbiol. 2022 Nov 10;13:1053583. doi: 10.3389/fmicb.2022.1053583 (PMC9686305; doi:10.3389/fmicb.2022.1053583)
Supplement: Supplementary file 1 [file Data_Sheet_2.pdf]

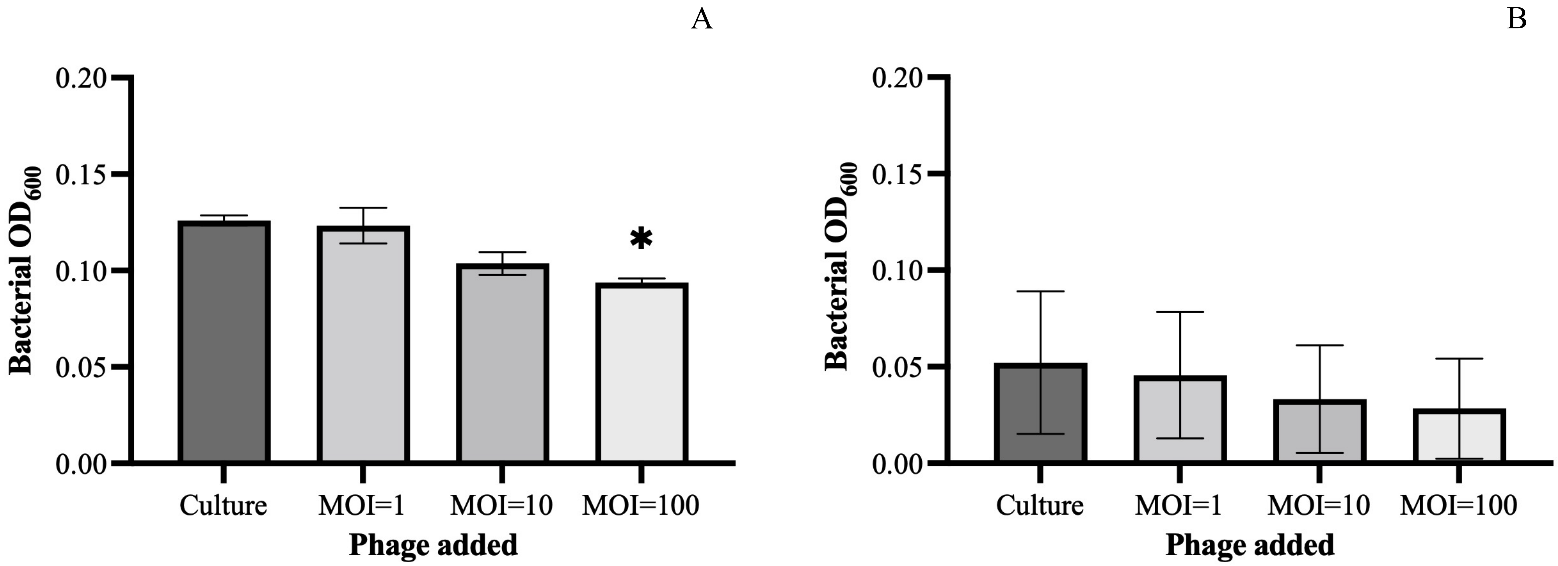

Supplementary Figure S1. Lysis from without of *E. coli* O157:H7 ATCC 35150 (A) and *Salmonella* Typhimurium ATCC 14028 (B) treated with phage Sa157lw at MOIs of 1, 10 and 100 at 25°C for 5 min based on the bacterial optical density at OD_{600}. Asterisk indicates a significant difference at $P < 0.05$. The error bars present the standard error of the mean (SEM) for each group.
